# Epidemics of Crimean-Congo Hemorrhagic Fever (CCHF) in Sudan between 2010 and 2020

**DOI:** 10.3390/microorganisms10050928

**Published:** 2022-04-28

**Authors:** Ayman Ahmed, Yousif Ali, Bashir Salim, Isabelle Dietrich, Jakob Zinsstag

**Affiliations:** 1 Institute of Endemic Diseases, University of Khartoum, Khartoum 11111, Sudan; 2 Swiss Tropical and Public Health Institute (Swiss TPH), CH-4123 Allschwil, Switzerland; jakob.zinsstag@swisstph.ch; 3 Faculty of Science, University of Basel, Petersplatz 1, CH-4001 Basel, Switzerland; 4 Sudanese National Academy of Sciences, Khartoum 11111, Sudan; 5 Health Emergencies and Epidemics Control General Directorate, Sudan Federal Ministry of Health, Khartoum 11111, Sudan; yousif.health@gmail.com; 6 Faculty of Veterinary Medicine, University of Khartoum, Khartoum 11111, Sudan; bashirsalim@gmail.com; 7 The Pirbright Institute, Pirbright GU24 0NF, UK; isabelle.dietrich@pirbright.ac.uk

**Keywords:** Crimean-Congo hemorrhagic fever (CCHF), zoonotic diseases, arboviral diseases, hemorrhagic fever, epidemic, outbreak, tick-borne diseases, climate change, neurotropic viruses, Sudan

## Abstract

Crimean-Congo hemorrhagic fever (CCHF) is a zoonotic arboviral disease that poses a great threat to global health in the Old World, and it is endemic in Europe, Asia, and Africa, including Sudan. In this retrospective study, we reviewed previous epidemiological reports about the major epidemics of CCHF throughout Sudan between 2010 and 2020. During these epidemics, the infection of humans with Crimean-Congo hemorrhagic fever virus (CCHFV), the causative agent of CCHF, was diagnosed using qRT-PCR. We have identified 88 cases of CCHF, including 13 fatalities reported during five epidemics that occurred in 2010, 2011, 2015, 2019, and 2020. The two epidemics in 2010 and 2011 were by far the largest, with 51 and 27 cases reported, respectively. The majority of cases (78%) were reported in the endemic region of Kordofan. Here, we document that the first emergence of CCHFV in the Darfur region, West Sudan, occurred in 2010. We were not able to investigate outbreak dynamics through phylogenetic analysis due to the limited diagnostic capacity and the lack of sequencing services in the country. These findings call for establishing a genomic-based integrated One Health surveillance and response system for the early preparedness, prevention, and control of CCHF in the country.

## 1. Introduction

The public health threat posed by arboviral diseases is rapidly growing worldwide and expanding its geographical distribution [1]. The majority of arboviral diseases are zoonotic, circulating among human and animal populations as they have evolved to continuously expand their host range through several spillover and spillback events [2]. The global risk of arboviral epidemics is increasing. This growth is influenced by several risk factors including globalization, unplanned urbanization, climate change, and socioeconomic inequalities [3,4,5,6]. Other risk factors include armed conflicts, largescale displacements of human populations between endemic and non-endemic areas, and humanitarian crises [7,8,9,10,11]. Arboviruses are emerging in under-resourced, tropical countries, where health systems are not prepared for their early detection and containment, and where limited disease surveillance capacity exists [4,12]. In these settings, arboviral infections are commonly misdiagnosed and treated as malaria [7,8,10]. Additionally, the lack of awareness of local healthcare providers and policymakers about the prevalence of arboviruses in their areas usually leads them to ignore these diseases in their differential diagnoses, surveillance, prevention, and health policy development [13]. The underreporting of arboviral diseases when accidentally detected is also common [4,13]. Moreover, the delay or entire dearth of timely sharing of epidemic data impacts global health, as the population at risk, healthcare providers, and public health policymakers are not alerted in time to take preventive measures [13].

Crimean-Congo hemorrhagic fever (CCHF) is a tick-borne viral infection that is caused by the Crimean-Congo hemorrhagic fever virus (CCHFV), which belongs to the genus *Orthonairovirus* in the *Nairoviridae* family [14]. CCHF is a zoonotic disease that infects both humans and animals. It commonly does not cause illness in animals, but it is life-threatening for humans, with high case fatality rates (CFRs) of up to 40% but also very high rates of asymptomatic infections [14,15,16]. According to the World Health Organization (WHO), CCHFV is the most predominant cause of viral hemorrhagic fever outbreaks worldwide [15]. CCHF is endemic in over 45 countries in the Old World, with human cases and epidemics reported in several countries in Africa, Asia, and Europe [15,16,17]. The WHO consider CCHF to be one of the top epidemic-prone diseases globally [15].

In addition to the location of Sudan, as a tropical country in an epidemic-prone region [18], it is endemic with several infectious diseases, including a wide range of vector-borne diseases such as malaria [19], Leishmaniasis [20], onchocerciasis [21], and many arboviral diseases [4]. This co-endemicity and co-transmission imposes a serious diagnostic and overall prevention and control challenge for the limited health system of the country [4]. Therefore, in the absence of a health policy for the prevention and control of arboviral diseases in Sudan, the frequency and intensity of arboviral disease outbreaks are persistently increasing. In addition, the distribution of these arboviruses is steadily expanding throughout the country [4,9,11]. This, in turn, has led to a remarkable shift in diseases’ burden, from malaria to arboviruses in endemic areas [3,22]. This switch in disease burden is mainly attributed to climate change; the prevalence of arboviral diseases is predicted to continue to grow worldwide over the next few decades [22]. More importantly, considering the zoonotic nature of these diseases, the epizootic transmission of arboviral diseases has a devastating socioeconomic impact on the local community and creates a great financial burden for governments in countries that rely on animals and their products [23]. CCHF is endemic in the central region of the country and has recently been reported in the Darfur area in West Sudan, where humans and livestock move freely across the open borders between Sudan, Libya, Chad, Central Africa, and South Sudan [4,8]. CCHFV was isolated from sheep and several species of ticks imported from Sudan to Saudi Arabia [24,25]. This highlights the serious role of the passive dynamics of tick vectors using domestic and wild animals, including birds as mobile vehicles, which eventually expands the geographic distribution of CCHFV-competent tick vectors, mainly members of the genus *Hyalomma* [26]. Numerous species of ticks, including several members of the genus *Hyalomma,* are distributed throughout Sudan, increasing the potential risk of CCHF emergence countrywide and expanding the geographical distribution of the disease beyond the endemic region of Kordofan [4,26]. This risk is further intensified by the reliance of the communities in this region on the production and export of animals and their products for human consumption nationally and internationally [27].

Here, we analyze major epidemics of CCHF in Sudan in 2010, 2011, 2019, and 2020, and discuss the implications of this virus’ transmission on the public health, food safety, and security in the region. Furthermore, we highlight the need for a One Health strategy to prevent and control future epidemics of CCHF and other zoonotic diseases.

## 2. Materials and Methods

This is a retrospective study analyzing data about CCHF epidemics that have occurred in Sudan. The data were extracted from outbreak investigation reports from the Ministry of Health. According to the national protocol for the investigation of viral hemorrhagic fever outbreaks, blood samples were obtained from suspected cases that were clinically identified with hemorrhages. Samples were shipped to the National Public Health Laboratory in Khartoum, where they were molecularly analyzed. The QIAamp viral RNA mini kit (Qiagen Inc., Hilden, Germany) was used for the extraction of total RNA from blood samples following the manufacturer’s instructions. Samples were tested for CCHFV using qRT-PCR Kits (Shanghai ZJ Bio-Tech Co., Ltd., Shanghai, China) following the manufacturer’s guidelines.

## 3. Results

**Figure 1:**
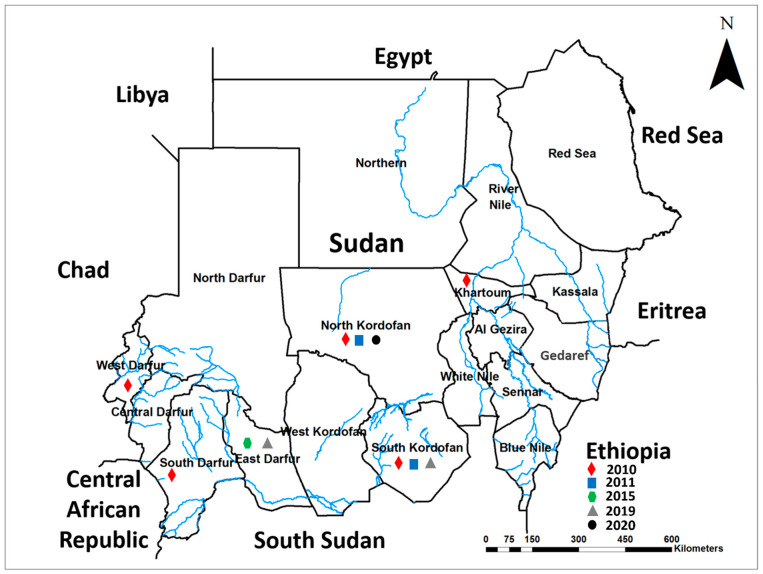
Geographical distribution of Crimean-Congo hemorrhagic fever (CCHF) epidemics in Sudan by years indicated by the colored shapes.

We have identified five epidemics of CCHF that occurred in Sudan during the previous ten years, between 2010 and 2020 (Figure 1). These epidemics occurred in 2010, 2011, 2015, 2019, and 2020 across several states of the country, namely Khartoum, North Kordofan, South Kordofan, East Darfur, North Darfur, South Darfur, and West Darfur states (Figure 1). However, the 2010 epidemic was the largest in magnitude and spread across five states (Figure 1).

**Figure 2:**
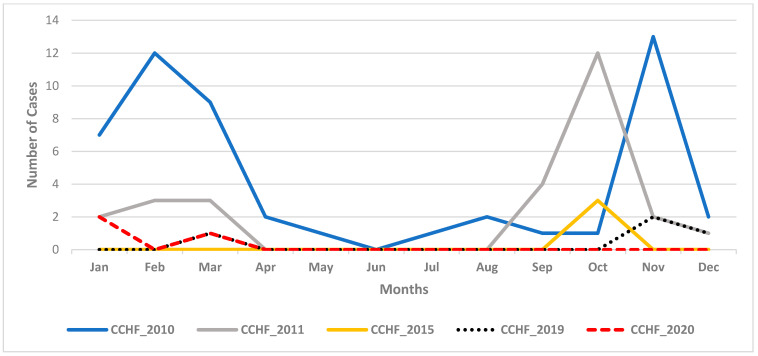
Epicurve showing Crimean-Congo hemorrhagic fever (CCHF) cases per month during the five epidemics between 2010 and 2020.

During these epidemics, 88 cases of CCHF in total were identified including 13 fatalities (Figure 2). Although cases were reported throughout the year during these epidemics, most cases clustered between September and January (Figure 2). Two major peaks developed during the two large epidemics in 2010 and 2011 between January and March, and September and November, while infections only clustered within one transmission season during the other epidemics in 2015, 2019, and 2020 (Figure 2).

**Figure 3:**
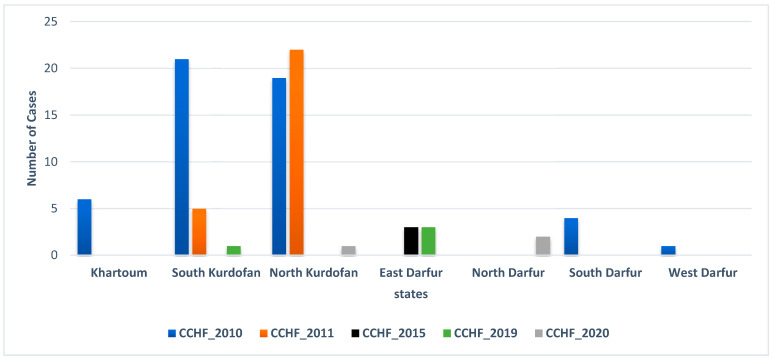
Number of Crimean-Congo hemorrhagic fever (CCHF) cases reported during each epidemic per state.

In the 2010 epidemic, 51 cases of CCHF including 12 deaths were reported in Sudan, of which 78% were from the Kordofan region, namely North and South Kordofan States (Figure 3). Six (12%) and five (10%) cases were reported from Khartoum State and the Darfur region, respectively (Figure 3). Interestingly, in 2010, CCHFV infections were reported for the first time from the Darfur region (Figure 3). Compared to the 2010 outbreak, the 2011 outbreak was less severe in terms of the number and severity of cases and the limited geographical distribution in the Kordofan region (Figure 3), with a total of 27 cases including a single fatality. Of these, 22 cases (81%) were reported from North Kordofan State, and 5 cases (19%) were reported from South Kordofan State (Figure 3). Three cases were reported in 2015 from East Darfur State. In the 2019 and 2020 epidemics, four and three cases were reported, respectively, distributed between the Darfur and Kordofan regions (Figure 3).

**Figure 4:**
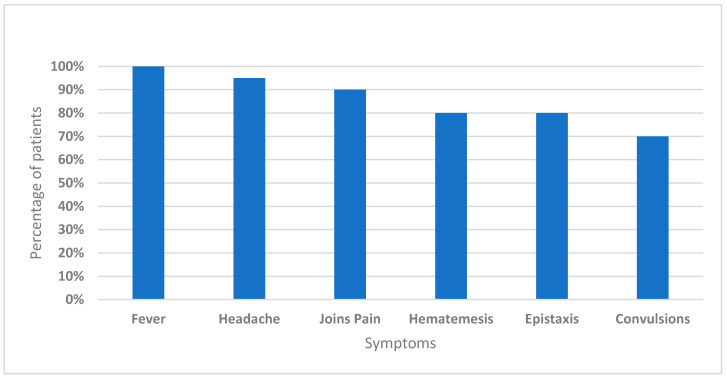
The clinical presentation of Crimean-Congo hemorrhagic fever (CCHF) cases.

Cases presented with fever (100%), headache (95%), and joint pain (90%), as well as with hematemesis and epistaxis (80%). Around 70% of the patients showed the involvement of neurological syndrome (convulsions) (Figure 4).

Twelve deaths were reported in 2010, representing a 24% case fatality rate (CFR), while a single death was reported in 2011 (CFR 4%). The overall male to female ratio of patients was 2:1, and the average age of patients was 28 years old. Cases presented with fever and hemorrhage, and no other clinical symptoms were recorded.

## 4. Discussion

In this report, we describe epidemics of CCHF that occurred between 2010 and 2020 throughout Sudan. We update the known distribution of CCHF in the country by reporting recent emergence events of CCHFV in the conflict and humanitarian environment of the Darfur region, West Sudan (Figure 1). The severe clinical presentations that were exhibited by patients included fever, headache, and joint pain, as well as hematemesis, epistaxis, and neurological syndrome (Figure 4). Very high rates of CCHF infections are inapparent, indicating that the original investigations of these epidemics have only identified the severe cases [14,15,16]. The involvement of neurological syndromes among patients with CCHF suggests that the pathogenesis of the disease in humans is beyond just liver manifestation and include the nervous system [28,29].

Here, we show that the geographical distribution of the diseases in Sudan is rapidly expanding out of the original endemic region of Central Sudan [4]. This could be attributed to the increase in the risk factors that influence the transmission of arboviral infections including climate change and humanitarian crises [4,30]. The severe weather events during the recent years in Sudan, including droughts, heavy rainstorms, and flooding, may have contributed to the spread of CCHF by influencing the dynamics of human and animal populations in and out of CCHF-endemic areas, carrying the vector and/pathogen passively [9,30]. In particular, a study implemented among the one-humped camel population in Khartoum state, a non-endemic area for CCHF, revealed a high seroprevalence of CCHF-IgG (over 21%), which is very alarming [31]. This finding suggested the serious role of animal dynamics in spreading zoonotic diseases, including CCHF [31]. Similar scenarios were observed with Chikungunya, dengue, and Rift Valley fevers [3,8,9,11]. CCHF causes severe epidemics of viral hemorrhagic fever with a high fatality rate. A 24% CFR was reported during the 2010 epidemic, yet the disease is severely neglected by the local healthcare providers, researchers, and public health policymakers. This neglect is mainly due to the lack of awareness about the serious health threats imposed by the disease [4,14,15]. This lack of awareness is further underscored by the absence of an early warning surveillance and response system, an adequate health policy for disease prevention and control, and support for research to fill the gaps in our knowledge about the disease [4,14]. Additionally, this underestimation of the public health risk of CCHF in the country is further intensified by the limited reporting and data-sharing culture among health authorities and the lack of animals used as mobile sentinel sites for early detection [13,32]. Therefore, the disease in Sudan commonly emerges in nosocomial outbreaks, risking the lives of healthcare providers [33,34]. The case fatality rate in 2010 was 24%, which is typical of the documented rate, while it was relatively low in 2011 at 4% [14,15]. The mortality rate was relatively high in naive areas such as South Darfur, with a 50% case fatality rate, and 33% in Khartoum, compared to the case mortality rate in cases from endemic areas of the Kordofan region (20%). This apparent reduction in cases per epidemic after 2011 could be attributed to the dysfunction of the health system in the endemic region of Kordofan due to the presence of armed conflict in the area [35,36]. Moreover, armed conflict in the Darfur region, Western Sudan, drastically changed the environment and the socioeconomic structure of the local communities, which, in turn, increased the vulnerability of poor communities with fragile healthcare to the emergence of infectious diseases, including arboviral infections [7,8,9,10,30]. Our investigation reveals that CCHF emerged for the first time in the Darfur region in 2010. Apparently, the disease has established endemicity in the area with cases of CCHF reported from the region in 2016 during an epidemic of febrile illness [4,8].

One of the main challenges undermining our understanding of the disease’s transmission, burden, and epidemiology in Sudan is the limited diagnostic capacity and disease surveillance system throughout the country, with the very limited use of molecular and serological diagnostic tools in healthcare facilities [4,12,13]. Additionally, the limited resources in the country and the lack of support for research are challenging investigation attempts to generate evidence to inform and guide the health system [4]. Nonetheless, limited studies showed that at least two strains of CCHFV (Sudan Al-fulah 2018, and Sudan Abyei 2009) are circulating in Sudan and were involved in previous separate epidemics [33,37]. Recent studies about the emergence of the invasive Asian malaria vector *Anopheles stephensi* in the country have highlighted the importance of using molecular and genomics sequencing tools for the early detection and monitoring of the disease’s vector dynamics [38,39,40].

Exposure to tick bites, animals living in close proximity, and handling sick animals and their infected products are the major risk factors for getting infected with CCHFV [14,15,16]. This leaves poor individual and farming-dependent communities at higher risk of the disease. In particular, the inapparent infection of CCHF among cattle could help spread the disease further, not only through the vectors, but through direct contact with infected animal products, leaving butchers and slaughterhouse workers at higher risk unless strict food safety measures including testing animals for infection and proper infection control measures are implemented [41]. Healthcare providers in endemic areas and/or attending to CCHF patients during epidemics are at a similarly high risk [42]. Particularly in limited diagnostic settings where CCHFV infections are not robustly detected, infections among healthcare providers are fatal [17,25,26,34]. The common delay in the detection of zoonotic diseases has an extremely high accumulative societal cost on the endemic communities [23]. Moreover, other professionals with an occupational hazard of CCHF include agricultural workers, slaughterhouse workers, and veterinarians [15,16]. Therefore, the implementation of an early-preparedness strategy with a One Health approach is crucial for the prevention and control of CCHF epidemics [23,43]. Zoonotic diseases in general have devastating economic impacts. It was reported in 2012 that the annual cost of zoonotic and food-borne diseases is approximately USD 84 billion, 60% of which is due to the cost of human health, 30% is attributed to animal mortality, and 10% to lost productivity [44]. Arboviral diseases pose a severe socioeconomic burden, particularly on poor farm-depending communities, yet those of CCHF are understudied [6,22,37,38]. Therefore, social studies investigating the societal impacts of CCHF and its outbreaks are needed to quantify the cost-effectiveness of prevention and control measures. In particular, safe treatment and vaccines for CCHF are still underdeveloped, and the currently recommended prevention and control measures include avoiding exposure to ticks and the use of personal protective equipment when handling patients and sick animals, specifically in endemic areas [15,16].

The emergence and growing spread of CCHF into novel areas in Sudan seems to be associated with the recently reported changes in biology and expansion of the geographical distribution of tick vectors of CCHFV [45,46]. In addition to their roles as the main vector for transmitting CCHFV, virus circulation in endemic areas is mainly maintained through vertical transmission in tick populations, also known as transovarial transmission from mother ticks to the next generation [26]. Figure 2 clearly indicates the seasonality of CCHF epidemics throughout the years, with a remarkable increase in human cases between mid-September and late March (Figure 2), which corresponds to the rainy season when animals are sent out into the open pastures with their caretakers [11]. A relatively recent study has revealed that cattle pasturing on open grassland were at 27 times the risk of CCHF, compared to cattle in closed farms [47]. The rainy season might also correspond to the increase in the abundance, distribution, and exposure to tick vectors of CCHFV; however, there is a severe gap in data about the seasonality and change in vector populations. Further studies about the distribution, bionomics, dynamics, and vector composition of ticks in the CCHF endemic areas are warranted to generate evidence data that are essential for establishing an effective vector control strategy [48]. This indicates that unless serious action is taken to prevent and control the disease, the threat of CCHF for both public health and the economy will persistently grow, locally and regionally.

Although some antiviral drugs such as ribavirin are promising and vaccines are under development, the current lack of treatments and safe vaccines for preventing CCHF infections urges the need for a vector focused prevention health policy [15,16]. An integrated One Health policy that considers the health of humans, animals, the environment and their co-dependent interactions would reduce the threats of arboviral diseases including CCHF. Such a health policy should aim to increase the awareness of the communities at risk and promote the use of personal protection equipment and clothes to avoid contact with ticks. It must also implement vector control measures for tick populations; invest in improving diagnostic capacity and early warning and response systems; and train healthcare providers for humans and animals on personal safety and infection control during the treatment [23].

## 5. Conclusions

The global health threat of CCHF is rapidly growing worldwide, including in Sudan, without notice from the healthcare providers and public health policymakers. An early warning system and improvement of diagnostic capacity, particularly in endemic and epidemic-prone areas, is crucial for reducing the frequency and intensity of disease outbreaks and their morbidity and mortality. An effective health policy and integrated One Health strategy for the control and prevention of zoonotic diseases including CCHF needs to be established with multisectoral coordination between the Ministry of Health, the Ministry of Animal Resources, and the Environmental and Metrological authorities and their partners with transparent and timely data sharing. We recommend the establishment of a disease control program for the early detection, prevention, and control of arboviral diseases and hemorrhagic fevers including CCHF. In addition, the Ministry of Health must invest substantial effort and resources in vector surveillance and control throughout the county. Incorporating the use of advanced molecular and genomic sequencing techniques will provide crucial information on the dynamics of pathogens and their vectors, which, in turn, will inform policymakers and guide the national strategy for disease control.

## Data Availability

All data used in this study are included in the published paper.
