# Correction: Ahmed et al. Epidemics of Crimean-Congo Hemorrhagic Fever (CCHF) in Sudan between 2010 and 2020. *Microorganisms* 2022, *10*, 928

**DOI:** 10.3390/microorganisms14030517

**Published:** 2026-02-24

**Authors:** Ayman Ahmed, Yousif Ali, Bashir Salim, Isabelle Dietrich, Jakob Zinsstag

**Affiliations:** 1 Institute of Endemic Diseases, University of Khartoum, Khartoum 11111, Sudan; 2 Swiss Tropical and Public Health Institute (Swiss TPH), CH-4123 Allschwil, Switzerland; jakob.zinsstag@swisstph.ch; 3 Faculty of Science, University of Basel, Petersplatz 1, CH-4001 Basel, Switzerland; 4 Sudanese National Academy of Sciences, Khartoum 11111, Sudan; 5 Health Emergencies and Epidemics Control General Directorate, Sudan Federal Ministry of Health, Khartoum 11111, Sudan; yousif.health@gmail.com; 6 Faculty of Veterinary Medicine, University of Khartoum, Khartoum 11111, Sudan; bashirsalim@gmail.com; 7 The Pirbright Institute, Pirbright GU24 0NF, UK; isabelle.dietrich@pirbright.ac.uk

In the original publication [1], the case numbers given in the main text did not match the data presented in Figure 2. The number of deaths was incorrectly presented. The resulting mortality rates were, thus, incorrect. The following corrections have been made to parts of the Results and Discussion, respectively:

In paragraph 2 of the “Results” section, the sentence “Another large epidemic was reported in 2011 with 27 cases reported exclusively from the Kordofan region” was deleted. The correct paragraph 2 is as follows:

During these epidemics, 88 cases of CCHF in total were identified including 13 fatalities (Figure 2). Although cases were reported throughout the year during these epidemics, most cases clustered between September and January (Figure 2). Two major peaks developed during the two large epidemics in 2010 and 2011 between January and March, and September and November, while infections only clustered within one transmission season during the other epidemics in 2015, 2019, and 2020 (Figure 2).

In paragraph 3 of the “Results” section, the first sentence was modified to “In the 2010 epidemic, 51 cases of CCHF including 12 deaths were reported in Sudan, of which 78% were from the Kordofan region, namely North and South Kordofan States (Figure 3).”

In paragraph 5 of the “Results”, the first sentence is modified to “Twelve deaths were reported in 2010, representing a 24% case fatality rate (CFR), while a single death was reported in 2011 (CFR 4%).”

Paragraph 2 of the “Discussion” section is updated as follows:

Similar scenarios were observed with Chikungunya, dengue, and Rift Valley fevers [3,8,9,11]. CCHF causes severe epidemics of viral hemorrhagic fever with a high fatality rate. A 24% CFR was reported during the 2010 epidemic, yet the disease is severely neglected by the local healthcare providers, researchers, and public health policymakers. This neglect is mainly due to the lack of awareness about the serious health threats imposed by the disease [4,14,15]. This lack of awareness is further underscored by the absence of an early warning surveillance and response system, an adequate health policy for disease prevention and control, and support for research to fill the gaps in our knowledge about the disease [4,14]. Additionally, this underestimation of the public health risk of CCHF in the country is further intensified by the limited reporting and data-sharing culture among health authorities and the lack of animals used as mobile sentinel sites for early detection [13,32]. Therefore, the disease in Sudan commonly emerges in nosocomial outbreaks, risking the lives of healthcare providers [33,34]. The case fatality rate in 2010 was 24%, which is typical of the documented rate, while it was relatively low in 2011 at 4% [14,15]. The mortality rate was relatively high in naive areas such as South Darfur, with a 50% case fatality rate, and 33% in Khartoum, compared to the case mortality rate in cases from endemic areas of the Kordofan region (20%). This apparent reduction in cases per epidemic after 2011 could be attributed to the dysfunction of the health system in the endemic region of Kordofan due to the presence of armed conflict in the area [35,36]. Moreover, armed conflict in the Darfur region, Western Sudan, drastically changed the environment and the socioeconomic structure of the local communities, which, in turn, increased the vulnerability of poor communities with fragile healthcare to the emergence of infectious diseases, including arboviral infections [7–10,30]. Our investigation reveals that CCHF emerged for the first time in the Darfur region in 2010. Apparently, the disease has established endemicity in the area with cases of CCHF reported from the region in 2016 during an epidemic of febrile illness [4,8].

The authors state that the scientific conclusions are unaffected. This correction was approved by the Academic Editor. The original publication has also been updated.
